# Development and characterization of DIA 12.3, a fully human intact anti-CEACAM1 monoclonal antibody

**DOI:** 10.1371/journal.pone.0295345

**Published:** 2024-02-12

**Authors:** Michela Centonze, Valentina Fiori, Maciej Kujawski, Lin Li, Patty Wong, Lindsay Williams, Tomas Di Mambro, Sabrina Dominici, Angelo Sparti, John E. Shively, Mauro Magnani

**Affiliations:** 1 Department of Biomolecular Science, University of Urbino, Urbino, Italy; 2 Diatheva Srl, Cartoceto (PU), Italy; 3 Department of Immunology and Theranostics, Beckman Research Institute, City of Hope, Duarte, California, United States of America; National Hospital Organization Minami Wakayama Medical Center, JAPAN

## Abstract

Carcinoembryonic antigen cell adhesion molecule-1 (CEACAM1), a homotypic cell adhesion molecule glycoprotein with apical expression on normal epithelial cells and activated lymphocytes, is overexpressed on many tumors and acts as an inhibitory receptor on NK cells, preventing their killing of CEACAM1 positive tumors. Production of humanized anti-CEACAM1 antibodies to block the inhibitory activity of CEACAM1 for immunotherapy and immunoimaging. Starting from a scFv, a fully human intact anti-CEACAM1 (DIA 12.3) that recognizes the N-terminal domain of CEACAM1 was developed and shown to bind CEACAM1 positive tumor cells and enhanced NK cell killing of CEACAM1 positive targets. DIA 12.3 bound to human neutrophils without activation, indicating they would be safe for human use. DIA 12.3 exhibited some cross-reactivity to CEACAM5, a tumor marker with high sequence homology to the N-terminal domain of CEACAM1. CEACAM1 PET imaging with ^64^Cu-COTA-DIA 12.3 showed excellent imaging of CEACAM1 positive tumors with reduced binding to CEACAM5 tumors. Based on its immunoinhibitory an immunoimaging activities, DIA 12.3 shows promise for therapeutic studies in man.

## Introduction

Carcinoembryonic antigen cell adhesion molecule-1 (CEACAM1), a homotypic cell adhesion molecule glycoprotein with apical expression on normal epithelial cells and activated lymphocytes, is overexpressed on many tumors making it an attractive target for antibody directed therapy [1]. However, the fact that the CEACAM gene family is large and that most CEACAM antibodies are directed to the highly homologous N-domain shared by all members of the family [2], it is important to demonstrate the specificity of CEACAM1 antibodies. In addition, since CEACAM1 is expressed as multiple splice forms [1], there is evidence that the long cytoplasmic main splice form, that includes two inhibitory ITIMs [1], may block target cell killing, especially in the case of NK cells that also express CEACAM1 [3]. Thus, a further requirement of therapeutic anti-CEACAM1 antibodies is that they can block this potentially negative effect on NK mediated tumor killing. Furthermore, since CEACAM1 is expressed on neutrophils [4, 5], the most populous leukocytes in circulation, there is concern that anti-CEACAM1 antibodies may cause their activation with deleterious side effects. Several murine monoclonal antibodies to CEACAM1 have been described. However, their therapeutic use is limited due to their inherent immunogenicity in man. Thus, the production of humanized anti-CEACAM1 antibodies is an important goal for clinical studies. In this respect, a humanized anti-CEACAM1 antibody, CM-24, that recognizes the N-terminal domain of CEACAM1, has recently entered clinical trials for solid tumors expressing CEACAM1 [6, 7]. DIATHIS1, a scFv antibody fragment also directed to the N-terminal domain of CEACAM1, was previously described and shown to enhance NK mediated cytotoxicity against melanoma cells [8]. In this work, we now describe the production and characterization of DIA 12.3 a fully human IgG1 version of the scFv DIATHIS1 antibody.

## Materials and methods

Animal studies were performed under protocol 91037 approved by the City of Hope Institutional Animal Care and Use Committee in accordance with the national Institute of Health Office of laboratory Animal Welfare Guidelines. Collection of discard blood was obtained from anonymous healthy donors at the City of Hope Blood Donor center (Duarte, CA, USA) under IRB 21108. No informed consent was required.

### Cells

HEK293 cells, metastatic bladder cancer cell lines (TCC-SUP and 5637) and Natural Killer (NK)-92 cells were from DSMZ (Germany). Metastatic melanoma cell line (MelC), originating from human metastatic melanoma specimens, was obtained from the Department of Therapeutic Research and Medicines Evaluation, Pharmacogenetics (ISS Rome, Italy). Metastatic colon cancer cell line (HT29) was obtained from Mario Negri Institute of Milan (Italy). Human breast cancer (MDA-MB-231) cell line was from ATCC. HEK 293 cells were cultured in DMEM medium supplemented with 10% of FBS (Sigma), 1mM L-glutamine (Carlo Erba), glucose and 1mM non-essential amino acids (Gibco). MelC and 5637 cells were cultured in RPMI 640 medium with 10% of FBS and 1mM L-glutamine. TCC-SUP cells were grown in DMEM medium with 20% of FBS and 1mM L-glutamine. NK-92 cells were cultured in α MEM medium with 10% FBS and 2mM L-glutamine and IL-2 (75 IU/ml, R&D system). HT29 cells were grown in McCoy’s medium with 10% FBS and 1mM L-glutamine. MDA-MB-231 cells were cultured in DMEM medium (Corning) with 10% of FBS (Sigma) and 1X GlutaMAX (Corning). All cells were grown at 37°C in a 5% CO_2_ humidified incubator.

### Construction of the bicistronic vector for the antibody expression

The encoding gene sequence of scFv Diathis LC and IgG1 HC were cloned into two separate pcDNA 3.1 (-) vectors (Genscript). The expression cassette bearing the CMV promoter, LC encoding gene and the polyA chain was amplified by PCR with the primers CMV BglII Fw and BGH PA NruI Rev, bearing the BglII and the NruI restriction site sequences, respectively. The amplified expression cassette and the vector bearing the IgG1 HC encoding gene, were digested by BglII (10U/μl) and NruI (10U/μl) restriction enzymes (Thermo Fisher). The digested insert and vector were ligated by T4 ligase (10U/μl, Thermo Fisher) and the ligation reaction was then transformed in E. coli DH5α competent cells. The bicistronic vector was mutagenized to introduce the N297A substitution, by using the GenArt Site-Directed Mutagenesis System (Thermo Fisher), according to the manufacturer’s instructions. PCR was performed on the plasmid DNA (20ng/ml) using the AccuPrime^™^ Pfx DNA Polymerase. The PCR product was then transformed in E. coli DH5α competent cells. The mutant transformed cell colonies were screened by High Resolution Melt (HRM) analysis and confirmed by sequencing.

### Development of the antibody-producing stable cell line

The day before the transfection, HEK 293 cells were seeded at the final density of 10x10^5^ cell/ml in DMEM medium. The bicistronic vector was linearized with ScaI (10U/μl, Thermo Fisher) for 4 hours at 37°C. The digested product was purified by the QIAquick PCR Purification kit (QIAgen). 1 μg of linearized DNA was mixed with Gene Cellin (EuroBio) and gently added to the cells. Following two weeks of G418 (Roche) antibiotic selection, supernatants from each cellular pools were screened by ELISA. The cellular pool with the highest absorbance value was diluted by limiting dilution method in DMEM with 0,5 μg/ml G418 in three 96 multiwell plates. After 15 days, the supernatants from each clone were tested by ELISA assay. The same procedure was applied on the highest antibody-producing clone to obtain the highest antibody-producing subclones.

### ELISA assay

Ninety-six-well plates (High Binding, Thermo Fisher) were coated with 100 μl/well of 1 μg/ml of human N-terminal + A1 constant Ig-like domain fragment of CEACAM1 antigen (DIATHEVA s.r.l.) diluted in PBS and incubated at 37°C for 16–17 hours. After washes with PBS containing 0,05% v/v of Tween-20 (TPBS), the plates were blocked with 150 μl/well of PBS containing 1% w/v of bovine serum albumin (PBSB) for 1 hour at 37°C. Upon TPBS washes, 100 μl of supernatant collected from the cellular transfected pools were added to the coated wells. For the antibody titration, the antibody was serially diluted in PBSB ranging from 100 to 0,00038 μg/ml and 100 μl/well of antibody dilutions were added to the wells. The plate was incubated for 90 minutes at 37°C. 100 μl/well of mouse anti-Human Fc antibody (Meridian) diluted 1:500 in PBSB were added to the wells and incubated 1 hour at 37°C. 30 minutes after the addition of 100 μl/well of ABTS substrate (Surmodics), the absorbance values were recorded at 405 nm on a microplate reader.

### Antibody purification by protein A affinity chromatography

The antibody-producing subclone was maintained in culture for 2 days at 37°C and 7 days at 30°C, starting from an initial density of 250.000 cells/ml. The antibody was purified from the collected supernatant by Protein A Affinity Chromatography using 1 ml MabSelect PrismA protein A resin (Cytiva). Cell supernatant was diluted 1:2 in phosphate buffer with 10mM EDTA pH 7.8 and loaded into the column and at 1ml/min flow rate. Column was washed with: 5mM EDTA phosphate buffer, 5mM EDTA phosphate buffer with 0.65M NaCl pH 7.8, 5mM EDTA phosphate buffer with 0.65M NaCl and 5% (v/v) IPA, pH 7.8. The antibody was eluted in 0.1 M Sodium citrate, 0.15M NaCl and 2% (v/v) glycerol (pH 3).

### SDS PAGE

Five μg of the antibody were diluted in Sample Buffer with or without ꞵ-mercaptoethanol to analyze the antibody at the reducing and non-reducing conditions, respectively. Samples were boiled for 5 minutes, loaded on a 10% of polyacrylamide gel and run at 100 V. The samples were visualized by staining with Coomassie brilliant blue.

### SEC-HPLC

Size Exclusion-High Performance Liquid Chromatography (SEC-HPLC) was performed with the HPLC Jasco LC-4000 system using a TSK Gel G3000SW_xL_L x I.D. column equilibrated with 0.1mol/L Na_2_SO_4_ + 0.05% NaN3 in 0.1mol/L Phosphate buffer, pH 6.7 at a flow rate of 0.5–1 ml/min. 100 μl of purified antibodies were injected into the column and to estimate its molecular wight, the system was calibrating by proteins of known molecular weight to build a standard curve: BSA 67,000 Da, Ovalbumin 43,000 Da, Ribonuclease 13,700 Da, Aprotinin 6,512 Da, Vitamin B12 1,355 Da. Results were analyzed by using the software ChromNAV Ver.2-Spectra Manager.

### Endotoxin level assessment

Limulus Amebocyte lysate (LAL) gel clot assay was performed by following manual’s instructions. Briefly, 1 ml of antibody dilutions in Endofree water were added to separate tubes containing the limulus amebocyte lysate. The tubes were incubated 30 minutes at 37°C without lids and then inverted 180°C. The antibody sample was considered negative for the presence of the endotoxins in absence of solid clots after the tube inversion.

### Flow cytometry analysis

MelC, HT29, 5637 and TCCSUP cells were plated at the final density of 5x10^5^ cell/ml. After washes, cells were incubated alone or with the antibody diluted in PBS with 1% w/v of BSA for 90 minutes at 37°C. Following the treatment, cells were washed and resuspended in PBS with 0.5% w/v of BSA and 1:1000 (v/v) diluted FITC goat anti-Human Fc secondary antibody (Abcam), and incubated for 1 hour at 37°C. Samples were washed and analyzed with a Flow Cytometer (Becton-Dickinson, NJ).

### Lactate Dehydrogenase (LDH) cytotoxicity assay

The cytotoxicity was evaluated with the LDH-Glo^™^ Cytotoxicity Assay kit (Promega). 2x10^5^ cells/mL of target tumor cells in basic DMEM with 5% v/v of FBS were incubated alone or in the presence of DIA 12.3 antibody for 30 minutes at RT. NK-92 cells were serially diluted ranging from 1x10^5^ to 1x10^3^ cells/mL in basic RPMI medium with 5% of FBS and seeded in triplicate. The target cell suspension was then incubated with the effector cells at increasing effector/target cell ratio ranging from 1:1 to 10:1 in triplicate, for 4 hours at 37°C. The supernatants from the wells were diluted in the LDH Storage buffer and incubated 1:1 with the LDH Detection Reagent at RT for 60 minutes. Luminescence was recorded from 30 to 60 minutes after the addition of the LDH reaction agent. The % of cytotoxicity was calculated as follows:

$$\begin{array}{l} \text{100}\times \left(\text{Experimental}\;\text{LDH}\;\text{Release}–\text{Medium}\;\text{Background}\right)/\\ \left(\text{Maximum}\;\text{LDH}\;\text{Release}\;\text{Control}–\text{Medium}\;\text{Background}\right)\;\text{.} \end{array}$$

### Human neutrophil isolation and enrichment

Fresh blood samples were obtained from anonymous healthy donors at City of Hope Blood Donor Center (Duarte, California). The blood was mixed with an equal volume of 0,9% NaCl (Cytiva) with 3% of dextran and incubated 20 minutes at RT. The leukocyte-rich plasma layer was centrifuged, the cell pellet resuspended in 0,9% NaCl and then layered above Hypaque-1077 (Sigma) and centrifuged 40 minutes at 400 x g at 20°C. After the hypotonic lysis of the RBC/neutrophil pellet in cold 0,2% NaCl for 20 seconds, the sample was enriched with neutrophils by using the EasySep Human Neutrophil Isolation kit (StemCell), according to the manual’s instructions. The purity of the enriched neutrophil population was assessed by flow cytometry using the APC-conjugated mouse monoclonal antibody against CD66b (Biolegend). In total, 10,000 events were recorded.

### Apoptosis detection of neutrophils

FITC Annexin V Apoptosis Detection kit with Propidium Iodide (PI) (Biolegend) was used for the apoptosis detection of neutrophils upon the incubation with or without 10 μg/ml of DIA 12.3 antibody in RPMI at 37°C for 0, 1, 2, 16, 18 and 20 hours. Neutrophils were then washed and resuspended in Annexin Binding Buffer at the final concentration of 1x10^6^ cell/ml. The cell suspensions were incubated with FITC Annexin V and Propidium Iodide Solution 15 minutes at RT in the dark. After the addition of the Annexin V Binding Buffer to each tube, the samples were analyzed by flow cytometry. In total, 10,000 events were recorded per each time point.

### Detection of neutrophil-released NO

Isolated neutrophils were resuspended at the density of 2x10^6^ cell/ml in RPMI medium and then serially diluted to 1x10^6^ cell/ml and 2x10^5^ cell/ml. CEACAM1-expressing MDA-MB-231 tumor cells resuspended at the density of 2x10^5^ cell/ml in RPMI medium. Neutrophil suspension was added to tumor cell suspension at 10:1, 5:1, 1:1 effector:target cell ratios, in duplicate. Different treatment conditions were tested: neutrophils or MDA-MB-231 cells were pre-treated with or without 10 μg/ml of DIA 12.3 for 30 minutes at 37°C and then co-incubated with MDA-MB-231 cells or neutrophils for 4 hours at 37°C; neutrophils and MDA-MB-231 cells were co-treated with or without DIA 12.3 for 4 hours at 37°C. Following the treatment, the Griess Reagent System (Promega) was used to detect NO_2_- released by neutrophils into the cell culture medium. Briefly, first Sulfanilamide Solution and then NED Solution were dispensed to all the wells, and the plate was incubated for 10 minutes at RT in the dark. The absorbance values from each well were recorded at 520nm within 30 minutes in a plate reader.

### Animal studies

Five x10^6^ cell/ml of parental, hCEACAM1 and hCEA-expressing MDA-MB-231 cell lines were harvested, washed twice with PBS and resuspended 1:1 in PBS matrigel (Corning). 500 000 cells from each cell line were injected into the mammary fat pad of eight week-old NOD.Cg-Prkdc scid Il2rg tm1Wjl/SzJ (NSG) female mice (Jackson Laboratory) (n = 3), previously anesthetized with isoflurane.

### Antibody conjugation to DOTA and radiolabeling with ^64^CuCl_2_

A 20-fold excess of DOTA-NHS (220 nmol, Macrocyclics, TX) in 4.5μl of H_2_O was added to 1.65 mg (11 nmol) of Dia12.3 antibody in 2.5 ml of PBS1X, and the pH was adjusted to 7.36 with 0.1N NaOH. The reaction solution was rotated at the RT under Argon overnight. Then the reaction mixture was dialyzed against the PBS 1X buffer with five times and sterile filtered. Dota conjugation was confirmed by Instant thin layer chromatogram (ITLC) performed by the Radiopharmacy Department at City of Hope. One hundred μg of DOTA-DIA 12.3 was radiolabeled with 10 mCi of ^64^CuCl_2_ in 0.1 M HCl at pH 4.5 by adjusting the pH with 0.2M ammonium acetate. The final product purified by Size-Exclusion Chromatography by the Radiopharmacy Department of City of Hope.

### PET imaging and biodistribution studies

Three weeks after establishment of MDA-MB-231 tumors, tumor-bearing NSG mice were injected with a single intraperitoneal injection of 1 mg in 0.1ml of PBS of intravenous immunoglobulin (IVIG). Two hours later they were injected via the tail vein with a single intravenous dose in the range of 113–120 μCi of 6,4 μg/mouse of ^64^Cu-DOTA-labeled DIA 12.3 with 100 μCi of product per 10 μg + 30 μg/mouse of unlabeled DIA 12.3 in 1% human serum albumin–buffered saline. Shortly before PET scanning, mice were anesthetized with isoflurane and then imaged in a prone position on a GNET PET/CT scanner (Sofie Biosciences) 44–48 hours p.i. Images were reconstructed as previously described (9) by using AMIDE software. Immediately after completion of the final scan, mice were sacrificed and dissected for biodistribution analyses. The tumor and various major organs were excised, weighted and assayed for radioactivity using an automatic calibrated gamma counter (Wizard). The percentage of injected activity dose per gram of tissue (%ID/g) was calculated for each specimen.

## Results and discussion

### Expression, purification and structural characterization of DIA 12.3 antibody

**Fig 1:**
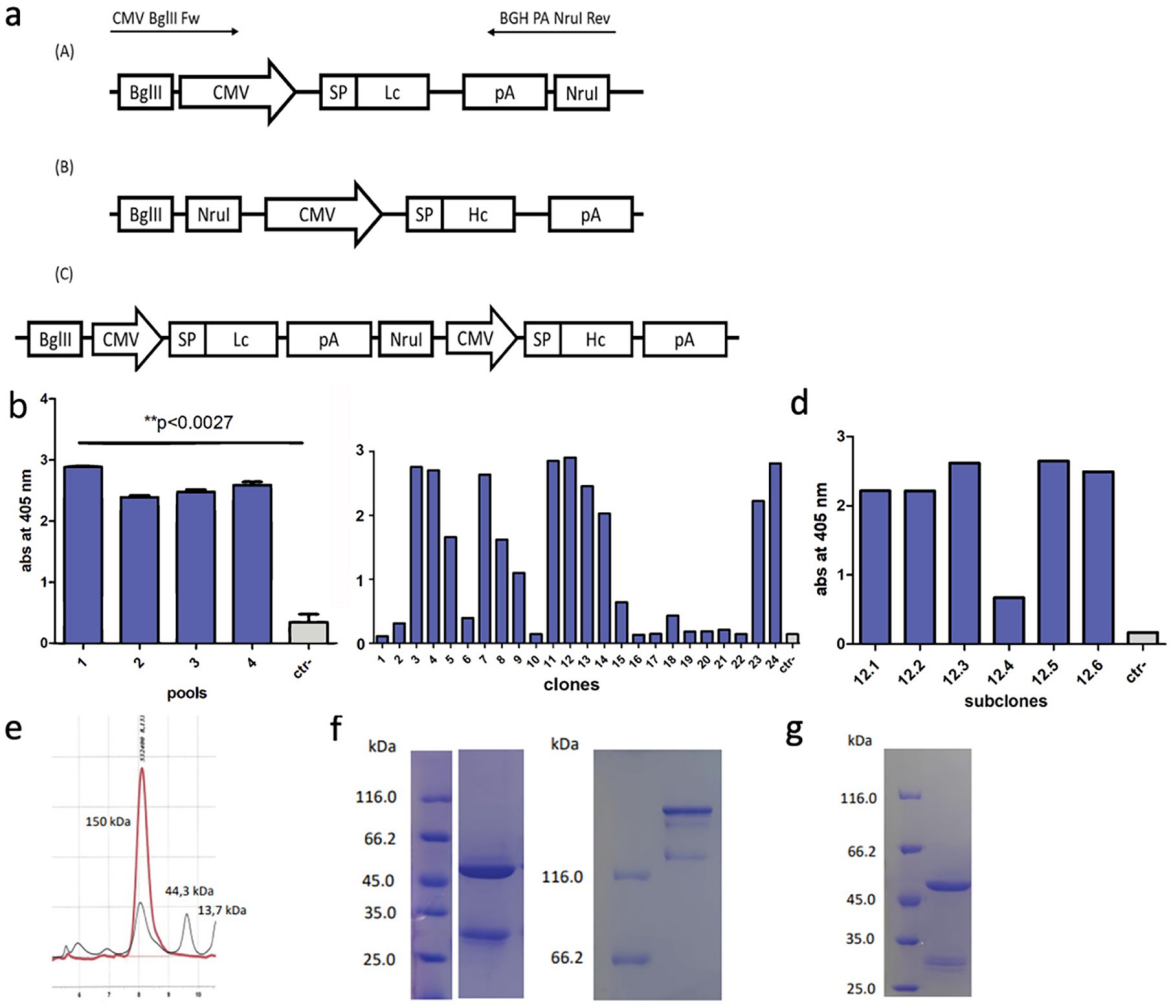
Development of the antibody-producing stable cell line and antibody characterization. **(a)** Construction of the bicistronic vector for the antibody expression. Three separate pcDNA 3.1 (-) vectors were used to clone the encoding gene sequences of scFvDiathis LC and IgG1 HC. The expression cassette bearing the CMV promoter, scFvDIATHIS1 LC encoding gene and the polyA chain, was amplified by PCR in order to add the sequence of BglII and NruI restriction sites, upstream and downstream the LC gene, respectively. The PCR product and the vector bearing the IgG1 HC encoding gene were both digested by BglII and Nru restriction enzymes. The digestion was followed by the ligation reaction to finally develop the bicistronic vectors for the simultaneous expression of the LC and the HC. Absorbance values obtained by ELISA assays on 100 μl of the supernatants collected from the transfected cellular pools **(b)**, clones **(c)** and subclones **(d)** for the production of the IgG1 antibody. The statistical significance of the pool differences in the abs values was tested with unpaired t-test. **(e)** SEC-HPLC chromatogram of IgG1 antibody purified from the subclone #12.3. The molecular weights were calculated based on a standard curve obtained by calibrating the system with molecules of known molecular weight: thyroglobulin 660 kDa, gamma- globulin 150 kDa, ovalbumin 44.3 kDa, ribonuclease A 43.7 kDa, 4-aminobenzoic acid 13.71 kDa. **(f)** Freshly purified antibody analyzed by SDS-PAGE under reducing (gel on the left) and non-reducing (gel on the right) conditions. **(g)** SDS- PAGE analysis of the antibody in reducing conditions after eight months of storage at 4°C.

In order to convert scFv DIATHIS1 into a fully intact IgG1 antibody, a bicistronic vector for the simultaneous expression of the Light Chain (LC) and the IgG1 Heavy Chain (HC) encoding genes, was created as illustrated in Fig 1a. In the final construct, the expression of the heavy and the light chain was driven by the CMV promoter. The N-terminal portion of the two genes was fused with a peptide signal sequence (SP) that enables the exportation of the four-chain antibody in the culture medium.

After two weeks of transfection of the HEK 293 cells with the bicistronic vector, the harvested cellular pools were tested with an ELISA assay for the antibody titer against the N-terminal + A1 domains (N+A1) of CEACAM1 (Fig 1b). The highest antibody-producing cellular pool was subjected to limiting dilution cloning. The highest expressing clone among the 24 IgG1-producing clones (Fig 1c) was sub-cloned by limiting dilution and the subclone #12.3 was identify by ELISA screening as the final stable cell line for antibody production (Fig 1d).

The antibody was purified from the cellular supernatant by Protein A affinity chromatography, resulting in a protein yield of 10.48 mg per 1L of supernatant. In order to purify a final product suitable for future therapeutic application, the purification process was optimized to reduce endotoxin contamination. All the glassware were treated with 1M NaOH for 2 hours. After washes with GMP water, the glassware was incubated at 250°C for 1 hour. All the purification buffers were prepared with GMP water and sterile filtered using filters with a 0.22μm pore size. Furthermore, to increase the antibody purity and further reduce the endotoxin contamination, the protein A chromatography protocol was modified by washing the column with phosphate buffer with 0.65M NaCl, followed by phosphate buffer with 0.65 NaCl and 5% (v/v) isopropanol (IPA). Prior to the antibody elution with sodium citrate at pH 3.0, an additional washing step with 0.1M sodium citrate at pH 5.0 was added to eliminate mAb fragments or unfolded mAbs that bind to protein A with lower affinity. This protocol resulted in a purified antibody with an endotoxin level lower than 10 EU/ml.

SEC-HPLC analysis revealed the expected molecular size of the antibody of 150 kDa with a high degree of purity, and a lack of molecular aggregates (Fig 1e). SDS-PAGE analysis under reducing conditions confirmed the expected molecular size of the heavy and the light chains of an IgG (Fig 1f). Despite the structural integrity highlighted by SEC-HPLC analysis, the SDS-PAGE under non reduced conditions revealed the presence of additional species of the antibody, such as dimers of the heavy chains (Fig 1f), suggesting incomplete formation of H-L disulfide bonds. The purified antibody exhibited structural stability by SDS gel electrophoresis after eight months of storage at 4°C (Fig 1g).

### Characterization of the antibody-producing stable cell line

**Fig 2:**
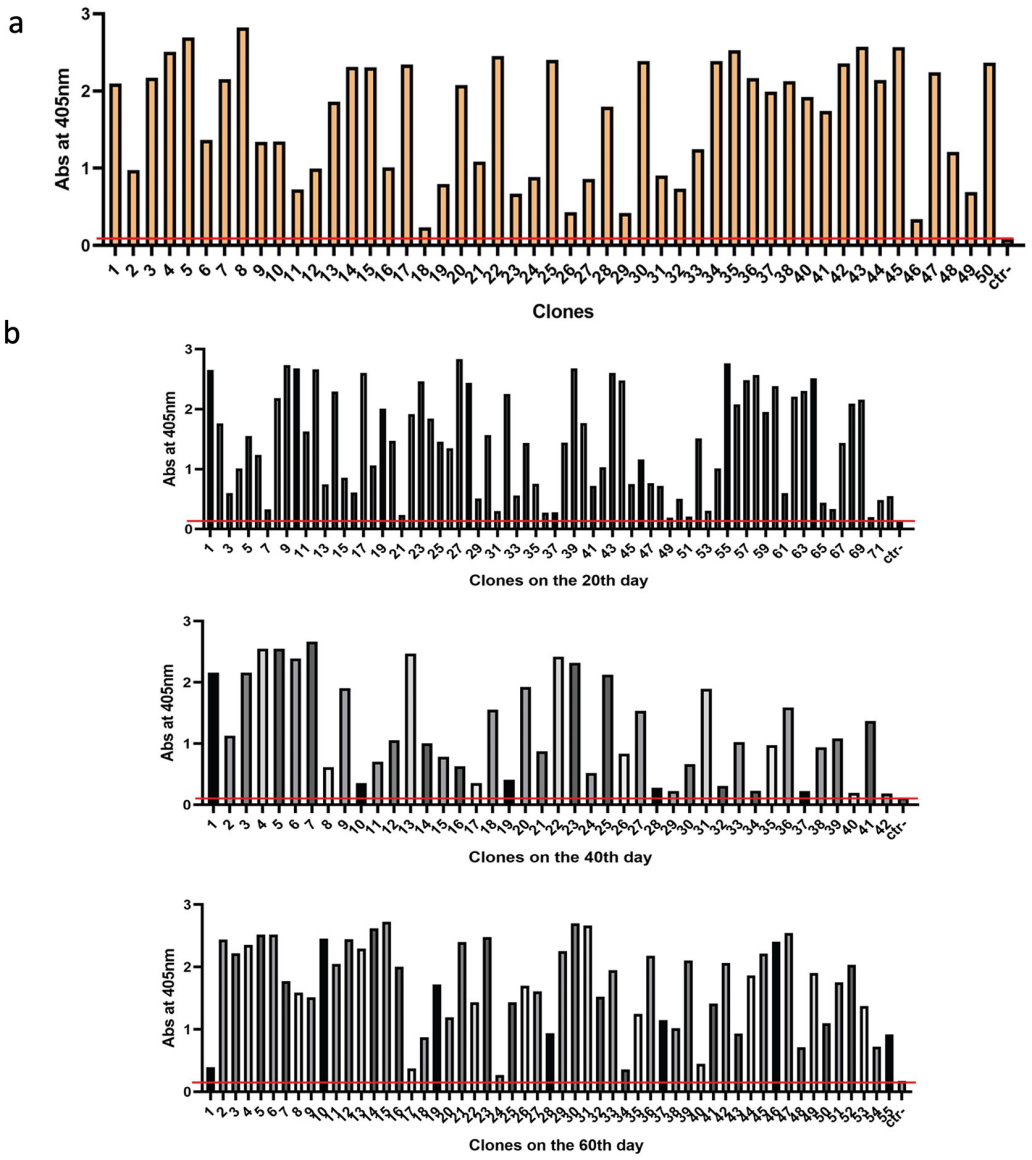
Characterization of the antibody-producing stable cell line. **(a)** Monoclonality of the subclone #12.3 is evaluated by ELISA assay. The graph shows the absorbance values of 50 clones grown 15 days after subjecting the subclone #12.3 to the limiting dilution. **(b)** Stability studies of antibody production from the subclone #12.3 by ELISA assay. The graphs report the absorbance values of the clones grown on the 20th, 40th and on the 60th day of culture without the pressure of the selective antibiotic.

The homogeneity of the DIA 12.3 antibody-producing cell line was confirmed by subjecting the subclone #12.3 to limiting dilution under the pressure of the selective antibiotic for two weeks. The grown clones were then tested in ELISA to evaluate the presence of cell subpopulations unable to secrete the antibody. All the clones were positive to the binding to the N+A1 domains of CEACAM1, confirming that the cell line is exclusively composed of antibody-producing cells (Fig 2a). The stability of the cell line for antibody expression was evaluated over prolonged periods of cell culture in absence of the selective antibiotic. The single cell cloning method was applied and ELISA assays were performed on the grown clones 15 days after each day of the limiting dilution, at 20th, 40th and 60th day (Fig 2b). However, plating the cells manually at a single cell density by limiting dilution might result in high variability in the number of arising clones at the different time points. Moreover, a variability in the clone size and cell numbers was observed, probably due to differences in the cell viability, affecting the mAb production by each clone. In particular, the absorbance values slight above the background signal were recorded for those clones composed of very few cells. Despite the variability in the clone size and the number of clone cells, results highlighted that more than 90% of the clones showed signals two-fold higher than the negative control at the tested times, suggesting there was no reversion to negative antibody producing cells during the 60 days of cell culture.

### DIA 12.3 and its mutant Fc mutant mtN297A DIA bind to CEACAM1 antigen expressed by tumor cells: DIA 12.3 but not the Fc mutant enhances the NK-mediated cytotoxicity

**Fig 3:**
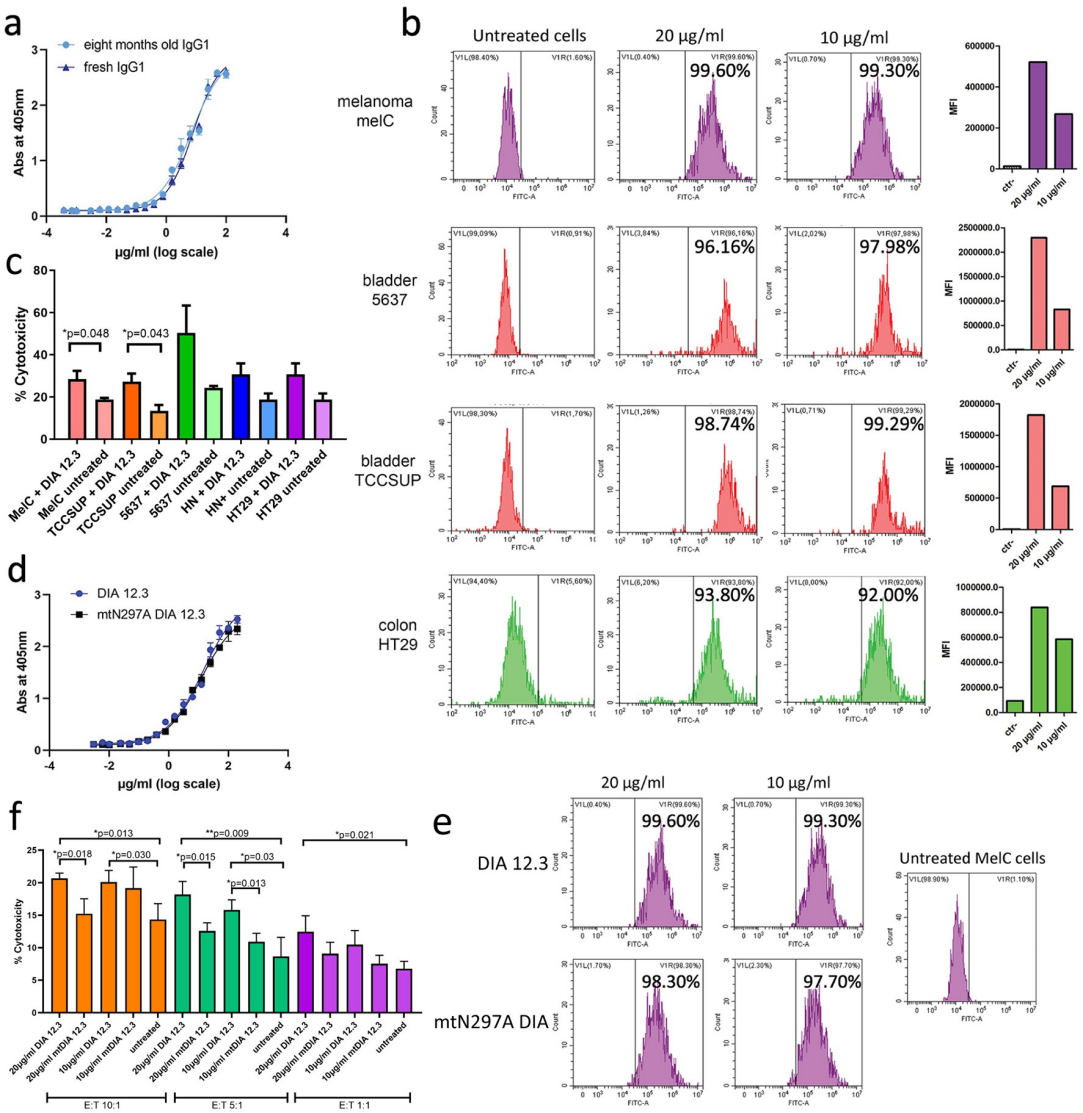
Functional characterization of DIA 12.3 and mtN297A DIA antibodies. **(a)** Binding activity of serial dilutions of DIA 12.3 antibody ranging from 100 μg/ml to 0,00038 μg/ml tested by ELISA titration assay on 96 multi-well plates coated with 1μg/ml human N+A1 domains of CEACAM1. The concentration values were log-transformed before performing the nonlinear regression on GraphPad Prism. Absorbance values are presented as mean +/- S.D of duplicate measurements. **(b)** DIA 12.3 binding analysis on tumor cells by flow cytometry: MelC, TCCSUP, 5637 and HT29 cells were treated with or without 20 and 10 μg/ml of DIA 12.3 antibody. The graphs showing the MFI values are reported on the right. **(c)** Cytotoxicity assay. MelC, TCCSUP and 5637, HT29 and Head&Neck cancer cells were treated with or without 20 μg/ml of DIA 12.3 antibody, and then co-incubated with NK cells at 10:1 E:T cell ratio. Luminescence was recorded after 60 minutes of incubation at RT. The plot is showing the % of NK cell-mediated cytotoxicity calculated upon antibody treatment in comparison with the basal NK cytotoxic activity against melanoma cells. The % of cytotoxicity was calculated as follows: 100 × (Experimental LDH Release–Medium Background)/(Maximum LDH Release Control–Medium Background). **(d)** and **(e)** Binding activity of mtN297A DIA in comparison with DIA 12.3 antibody by ELISA titration and flow cytometry assays. Serial dilutions of DIA 12.3 and mtN297A DIA antibodies ranging from 100 μg/ml to 0,00038 μg/ml were tested on 96 multi-well plates coated with 1μg/ml human N-terminal + A1 domain fragment of CEACAM1 antigen. The concentration values were log-transformed before performing the nonlinear regression on GraphPad Prism. Absorbance values are presented as mean +/- S.D of duplicate measurements. In the flow cytometry assay: MelC cells were treated with or without 20 and 10 μg/ml of DIA 12.3 or mtN297A DIA antibodies. **(f)** Cytotoxicity assay: MelC cells were treated with or without 20 or 10 μg/ml of DIA 12.3 antibody, and then co-incubated with NK cells at 10:1, 5:1 and 1:1 E:T cell ratios. Luminescence was recorded after 60 minutes of incubation at RT. The plot shows the percent of NK cell-mediated cytotoxicity calculated upon antibody treatment in comparison with the basal NK cytotoxic activity against melanoma cells. The percent cytotoxicity was calculated as described in Fig 2.

To evaluate the antigen binding activity of DIA 12.3, serial dilutions were tested by ELISA titration on the human N+A1 domains of CEACAM1. Results showed that the antibody binds the antigen in a dose-dependent manner and preserves the binding activity after eight months of storage at 4°C (Fig 3a). In addition, flow cytometry results confirmed that DIA 12.3 had a dose-dependent binding to CEACAM1 at the cell surfaces of melanoma, bladder, and colon cancer cell lines (Fig 3b).

Previous studies demonstrated that tumor cells exploit the homophilic interactions via the N+A1 domains of CEACAM1 on tumor cells and CEACAM1 on NK cells to escape from NK cell–mediated killing [3]. Thus, we investigated the ability of the DIA 12.3 to block the homophilic interactions and to rescue the NK cytotoxic activity against tumor cells. Antibody Dependent Cellular Cytotoxicity (ADCC) was evaluated by quantifying the LDH enzymatic activity in the cell culture supernatants. Following the treatment with DIA 12.3 antibody, melanoma, head and neck, bladder and colon cancer cells were co-incubated with NK cells at 10:1 effector:target (E:T) cell ratio. The calculated percent of cytotoxicity demonstrated that DIA 12.3 enhances the NK cell-mediated cytotoxicity against all the tested tumor cell lines (Fig 3c).

Although reduced NK mediated cytotoxicity has been ascribed to inhibitory homophilic interactions of CEACAM1 between tumor cells and NK cells [3], the mechanism of enhanced cytotoxicity by anti-CEACAM1 antibodies is an important issue since the NK FcR receptor CD16 is expected to play a role. Therefore, the well described N297A mutation [9] was introduced in the Fc region of DIA 12.3 to reduce the Fc effector functions. ELISA and flow cytometry assays revealed that the N297A mutation does not affect the mutated antibody binding activity to the N+A1 domains of human CEACAM1 (Fig 3d and 3e). However, the cytotoxicity assay showed that compared to DIA 12.3, the Fc mutated version had reduced NK cell mediated cytotoxicity similar to untreated controls (Fig 3f). in contrast, scFv DIATHIS1, that lacks an Fc domain, was able to enhance NK mediated cytotoxicity of double positive CEACAM1 target /NK cells [10], demonstrating a role for Fc/FcR NK cell interactions for DIA 12.3.

### DIA 12.3 binds to CEACAM1 on neutrophils without induction of apoptosis

**Fig 4:**
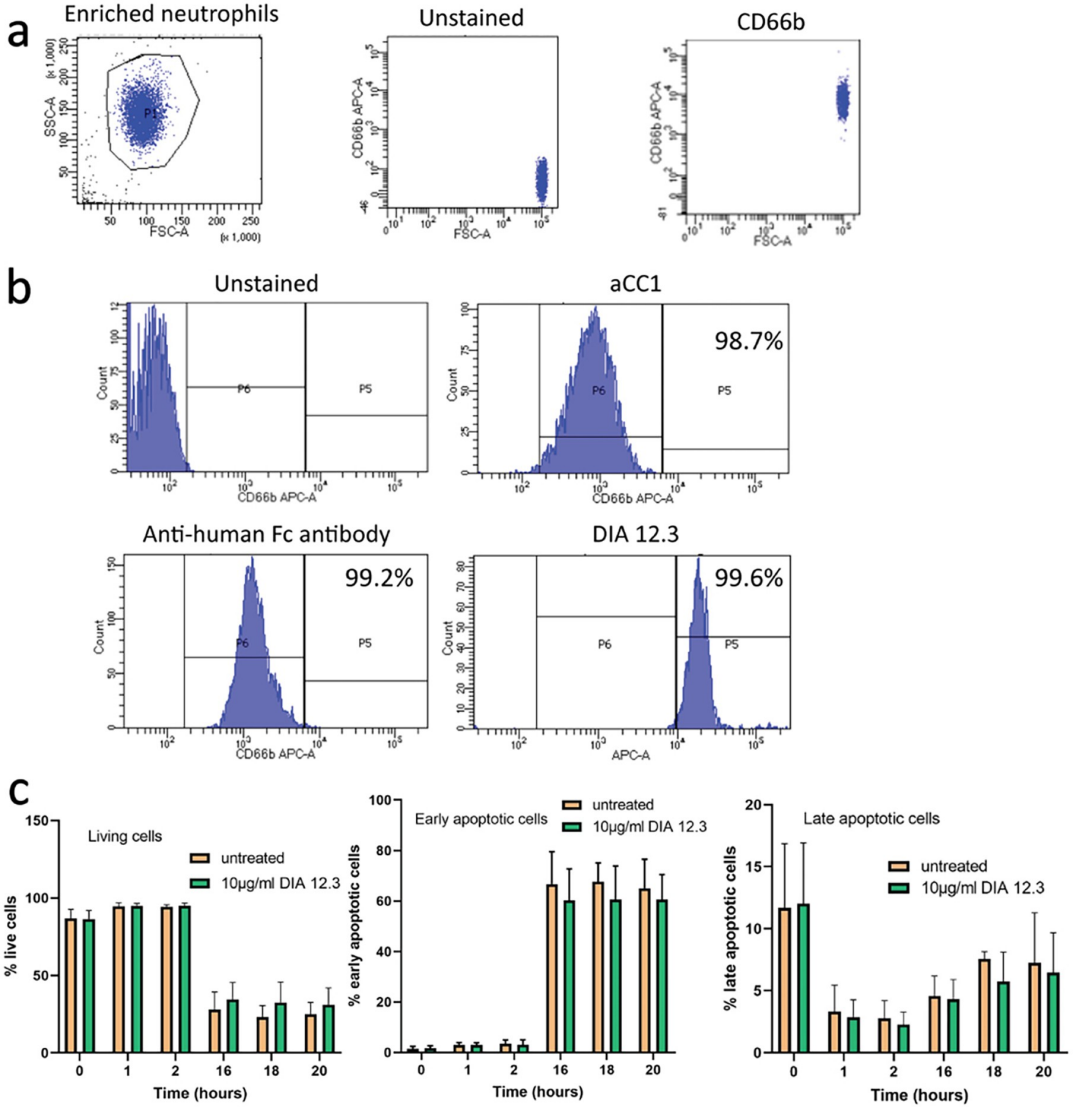
Effect of DIA 12.3 on spontaneous apoptosis ofneutrophils. (**a**) Neutrophil identification by CD66b staining after the isolation and (**b**) the binding profile of 10 μg/ml of DIA 12.3 in comparison with the commercial anti-CEACAM1 antibody. (**c**) spontaneous apoptosis in human neutrophils after DIA 12.3 treatment was evaluated. Neutrophils were cultured for 0, 1, 2, 16, 18 and 20 hours in RPMI medium with or without 10μg/ml of DIA 12.3 antibody at 37°C. At the end of each time point, neutrophils were double stained for FITC-annexin V and PI. Results show the % of early apoptotic, late apoptotic and living cells of three independent experiments. Bars are representative of the mean of triplicate values +/- SD.

Prior to clinical use, demonstration of the lack of potential toxic effects of anti-neutrophil antibodies is required. Flow cytometric analysis was used to assesses the purity of neutrophils isolated from healthy donors’ fresh blood after the enrichment procedure (Fig 4a). Staining with antibody against CD66b, used as surface marker for neutrophil identification, revealed the high purity of the neutrophil cell suspension. Results also showed that DIA 12.3 antibody recognizes and binds CEACAM1 expressed on cell surface of more than 96% of the neutrophil population, similar to a commercial anti-CEACAM1 antibody (Fig 4b). However, the addition of a secondary antibody that interacts with Fc receptors on neutrophils results in the observed higher binding.

To test the potential toxicity of DIA 12.3 antibody to neutrophils, the spontaneous neutrophil apoptosis was evaluated at 0, 1, 2, 16, 18 and 20 hours after the treatment with or without DIA 12.3 (Fig 4c). No positive antibody control was included in the apoptosis assay, since anti-neutrophil antibodies do not activate or induce apoptosis of neutrophils without prior priming [11]. As a result, we used the well-known spontaneous rate of neutrophil apoptosis as a control. Double-staining (annexin V-positive, PI-positive) revealed that the majority of neutrophils were early apoptotic cells after 16 hours of incubation at 37°C. The percent of late apoptotic cells (annexin V-positive, PI-negative) also increased after this period, concomitantly with the reduction of cell viability. Notably, a higher number of late apoptotic cells at time 0 compared to the following time points was observed, suggesting that the incubation of neutrophils in RPMI medium at 37°C might exert a positive effect on the viability of enriched neutrophils. Importantly, the results also showed that the rate of living, as well as apoptotic cells, was not altered after the treatment with DIA 12.3 antibody compared with the untreated samples, suggesting that the antibody binds to neutrophils without interfering with spontaneous apoptosis, a well know feature of neutrophils in culture.

### DIA 12.3 treatment does not affect neutrophil activation

**Fig 5:**
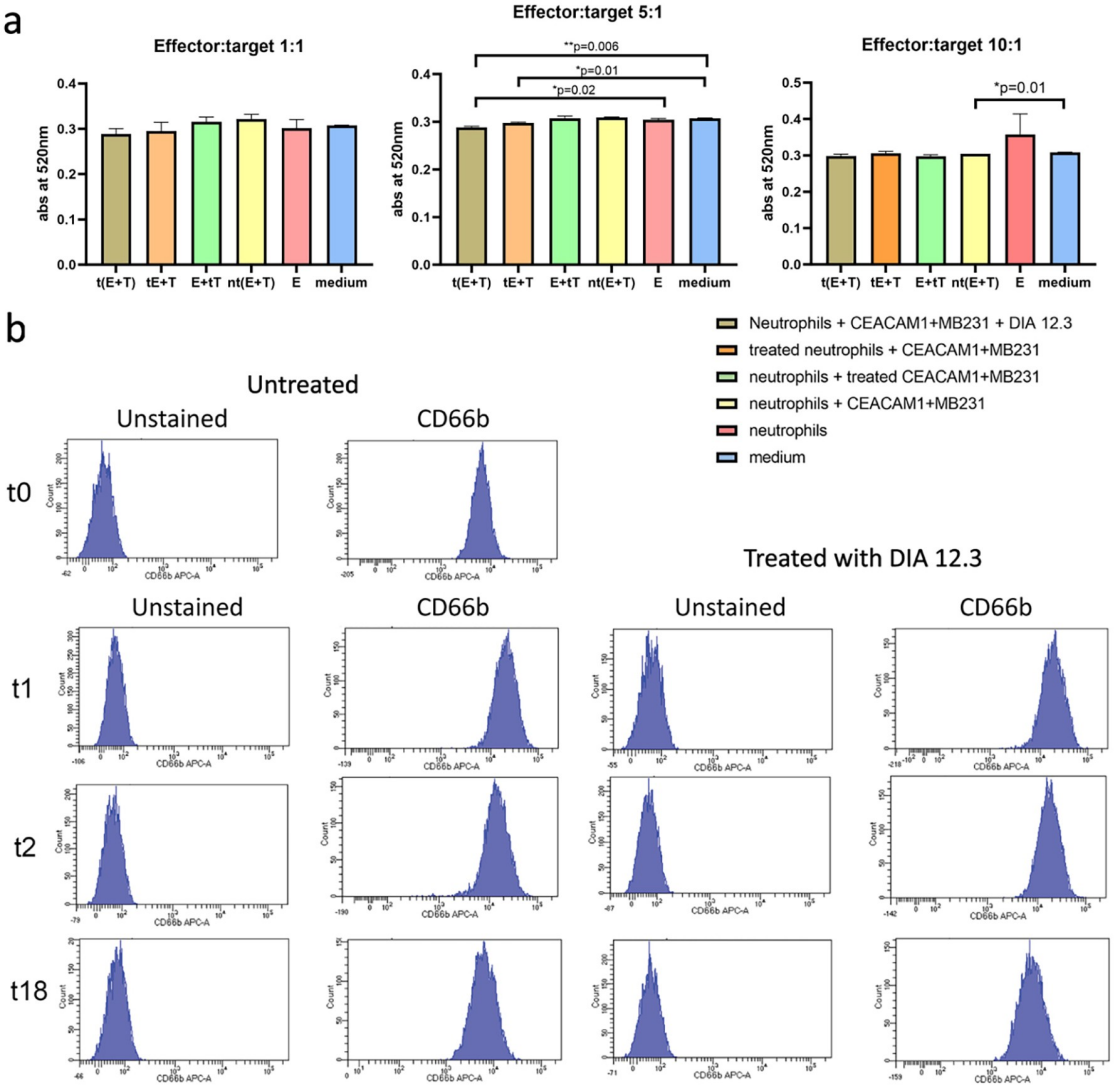
Neutrophil activation studies. (**a**) Analysis of NO levels released by neutrophils in culture supernatants. Neutrophils were co-cultured with CEACAM1 positive MB231 cells at 10:1, 5:1 and 1:1 E:T ratios. For each E:T cell ratio, three different treatment conditions were tested: (i) neutrophils or (ii) tumor cells alone pre-treated with or without 10 μg/ml of DIA 12.3 for 30 minutes at 37°C and then co-incubated 4 hours at 37°C with tumor cells or neutrophils, respectively; neutrophils and tumor cells co-treated with or without 10 μg/ml of DIA 12.3 for 4 hours at 37°C. Then the supernatants were subjected to nitrite assay according to the Griess reagent system. Bars are representative of duplicate values +/- SD. ** = P<0.01. (**b**) Evaluation of DIA 12.3 antibody effects on neutrophil activation. Isolated and enriched human neutrophils were incubated for 1, 2 and 18 hours in RPMI medium at 37°C in absence or presence of 10 μg/ml of DIA 12.3 antibody. Following each time point, neutrophils were then stained with anti-CD66b antibody.

To assess if DIA 12.3 antibody triggers NO production, a well-studied neutrophil-mediated mechanism that limits tumor progression [12], antibody treatments were performed on neutrophils co-cultured with CEACAM1-expressing breast tumor cell line MDA-MB-231 at different E:T cell ratios (Fig 5a). Neutrophil-produced NO was indirectly evaluated by measuring the concentration of nitrite, a stable and nonvolatile product of NO released by neutrophils in the supernatants, according to the Griess reaction. No statistically significant differences in the absorbance values were observed between the untreated neutrophils and the negative control (only medium) for none of the tested E:T cell ratios, confirming the lack of NO production by unactivated neutrophils. Furthermore, no alteration in the absorbance levels following the incubation with the antibody was observed, suggesting that DIA 12.3 antibody does not induce neutrophil-mediated NO production. These findings were in line with that observed in which neutrophils were treated with antibody against the activation marker CD66b. Neutrophils were CD66b positive soon after the enrichment protocol (t = 0, Fig 5b) and at 1, 2 and 18 hours after the incubation with or without the antibody, suggesting that the steps during isolation and enrichment procedures might activate neutrophils. However, treatment of neutrophils with DIA 12.3 did not lead to increased activation compared with the untreated samples, confirming that the antibody does not interfere with the physiological cellular processes of neutrophils. Interestingly, an increased level of activation was observed after 1 and 2 hours of incubation in the culture medium at 37°C, regardless of antibody treatment, concomitantly with the previously observed reduction of the percent of late apoptotic cells.

The investigation of neutrophil spontaneous apoptosis and their activation following antibody treatment of unactivated neutrophils, confirmed the safe applications of the antibody with neutrophils. In fact, the antibody does not induce toxic effects on neutrophils nor trigger possible neutrophil-mediated cytotoxic side effects due to their uncontrolled activation. These preliminary results will be followed by the in vitro efficacy experiments to test the efficacy of DIA 12.3 antibody to alter the CEACAM1 antigen-restricted tumor killing mediated by activated neutrophils.

### In vivo binding specificity of DIA 12.3 to hCEACAM1-expressing tumors

**Fig 6:**
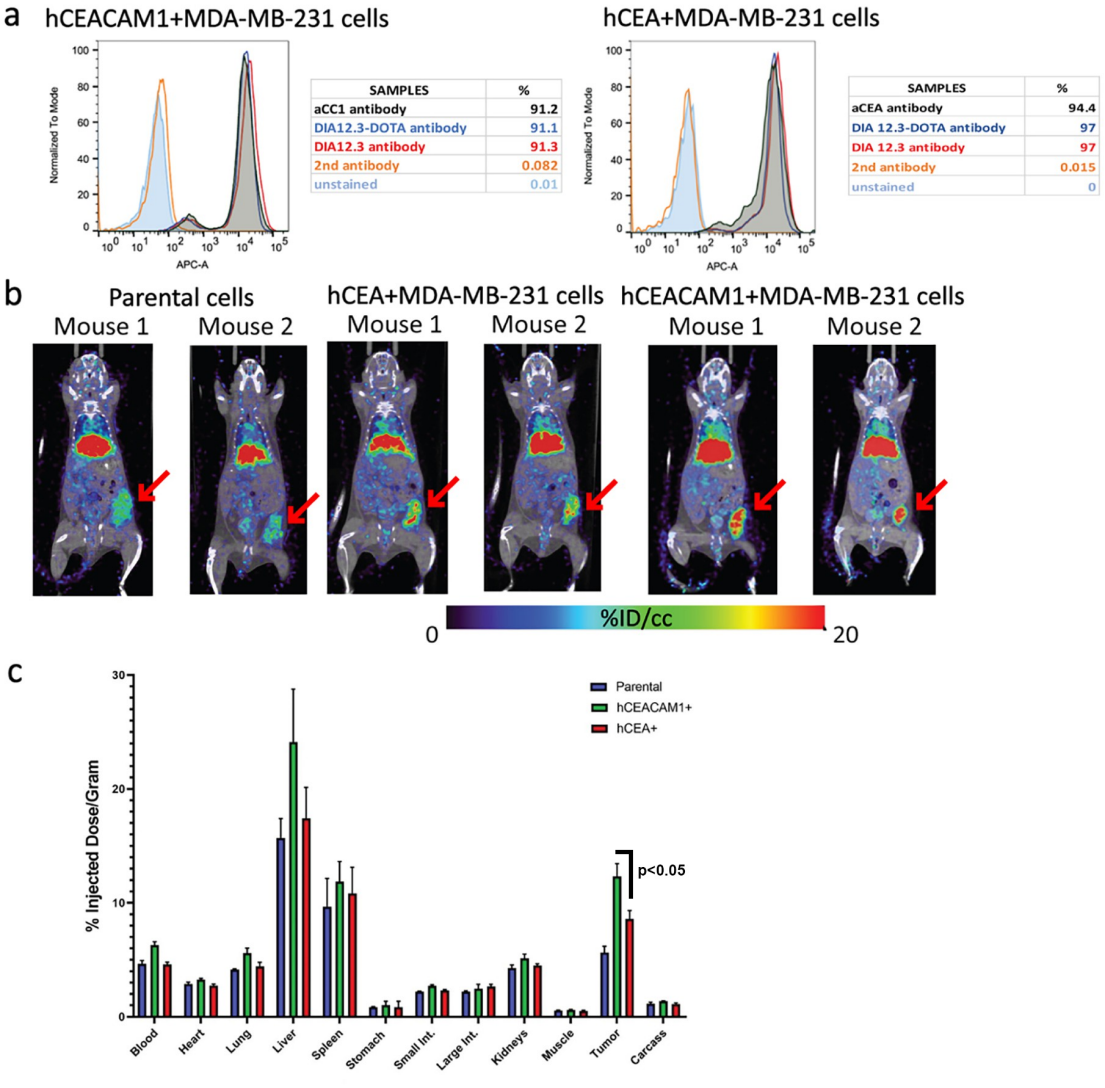
PET tumor imaging with ^64^Cu-DOTAylated-DIA 12.3 antibody in NSG mice bearing parental, hCEACAM1 and hCEACAM5-positive human breast tumor xenografts. (**a**) Binding profiles of DIA 12.3 and DOTAylated-DIA 12.3 antibodies on MB231 cell lines. hCEACAM1 and hCEA-expressing MB231 cells were incubated with or without 10 μg/ml of DIA 12.3 and DOTAylated-DIA 12.3 antibodies, anti-CEACAM1 or anti-CEA commercial antibodies. **(b)** PET images of mice bearing parental, hCEACAM1 or hCEA-positive human breast tumor MDA-MB-231 cells at 46 hours after the via tail vein injection of 6,4 μg/mouse antibody radiolabeled with 3.7 Mbq of ^64^Cu-DOTAylated-DIA 12.3 antibody + 30 μg/mouse of unlabeled DIA 12.3. Arrows indicated location of tumors. **(c)** Biodistribution analysis of the radiolabeled antibody 46 hours post-injection. Bars represent the mean +/- S.D. of the %ID/g in various organs and tumors from mice injected with each cell line (n = 3 mice per cell line).

In order to test the in vivo targeting of DIA 12.3 to CEACAM1 and CEACAM5 positive tumors, DIA 12.3 was conjugated to the chelate DOTA and radiolabeled with the positron emitter ^64^Cu, as previously described [13]. Flow analysis on breast tumor cells confirmed that the DOTAylation did not affect the binding ability of the antibody to either CEACAM1 or CEACAM5 (Fig 6a). Radiolabeling of DOTAylated-DIA 12.3 antibody gave a product that was 96.32% labeled with ^64^Cu radioisotope. To evaluate the antibody tumor-targeting, antigen-negative (parental), hCEACAM1 negative or hCEACAM5 positive human breast orthotopic tumor xenografts were established in mammary fat pads of female NSG mice. PET images of mice at 46 hours post-injection showed higher uptake levels of the antibody in the antigen-positive tumors compared to the parental tumor (Fig 6b). Furthermore, results from biodistribution analysis displayed that the % ID/g in hCEACAM1-positive tumors was about 12.5 ±0.4% compared to 7.9 ±0.3% (p<0.05) in the hCEA-expressing tumors at the same time point (Fig 6c), demonstrating the higher *in vivo* binding specificity of DIA 12.3 to hCEACAM1-positive tumors. While the high uptake in the liver and spleen is in accordance with the usual clearance mechanism of antibodies, the higher uptake in the liver for the CEACAM1 positive tumors may be due to clearance of soluble CEACAM1 observe with some tumors [14].

## Conclusion

Starting from a scFv, an all human IgG1 anti-CEACAM1 monoclonal antibody (DIA 12.3) was generated and expressed from a stable cell line. DIA 12.3 had high binding to an N-terminal fragment of CEACAM1 (N+A1 domains) in an ELISA and to a number of cancer cell lines known to express CEACAM1. DIA 12.3 exhibited potent cytotoxic activity against several cell lines in an NK assay demonstrating that it was able to overcome the inhibitory effect of CEACAM1 on NK cells. Mutation of a critical residue in the Fc domain, reduced cytotoxicity in an ADCC assay, showing that the Fc region was functional and played a role ADCC. PET imaging of radiolabeled DIA 12.3 demonstrated targeting to a CEACAM1 positive vs a negative cell line, as well as increased targeting to CEACAM1 vs CEACAM5 cell lines. Overall, this study demonstrates that DIA 12.3 may have good clinical utility.

## Supporting information

S1 Raw images Photo of original gel taken from Fig 1f. Lanes were cut and pasted into figure.
(PDF)
