# Supplementary material for: Development and characterization of DIA 12.3, a fully human intact anti-CEACAM1 monoclonal antibody
Source: PLoS One. 2024 Feb 12;19(2):e0295345. doi: 10.1371/journal.pone.0295345 (PMC10861082; doi:10.1371/journal.pone.0295345)
Supplement: S1 Raw images — Lanes were cut and pasted into figure. (PDF) [file pone.0295345.s001.pdf]

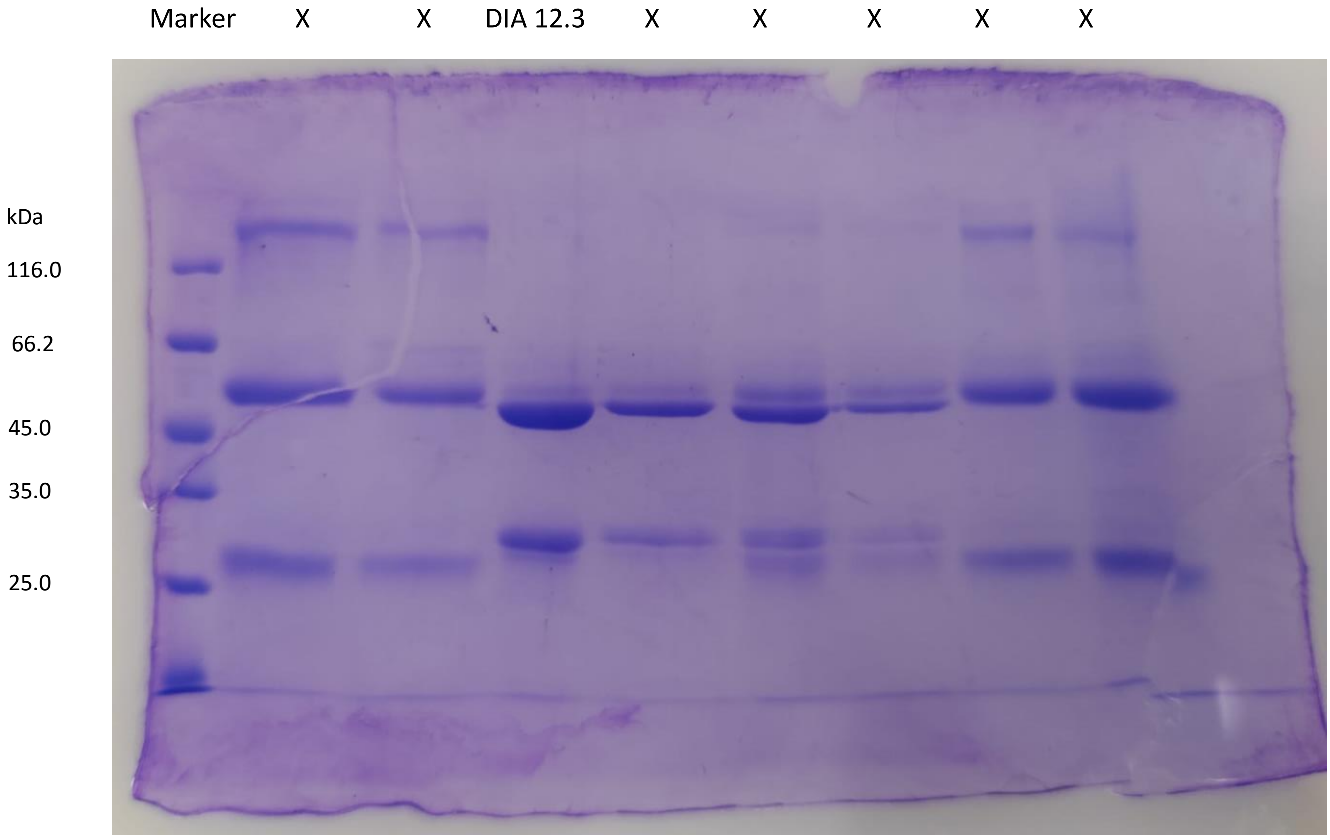

Fig 1f: SDS-Page under reducing conditions. The image was taken from the cell phone camera.

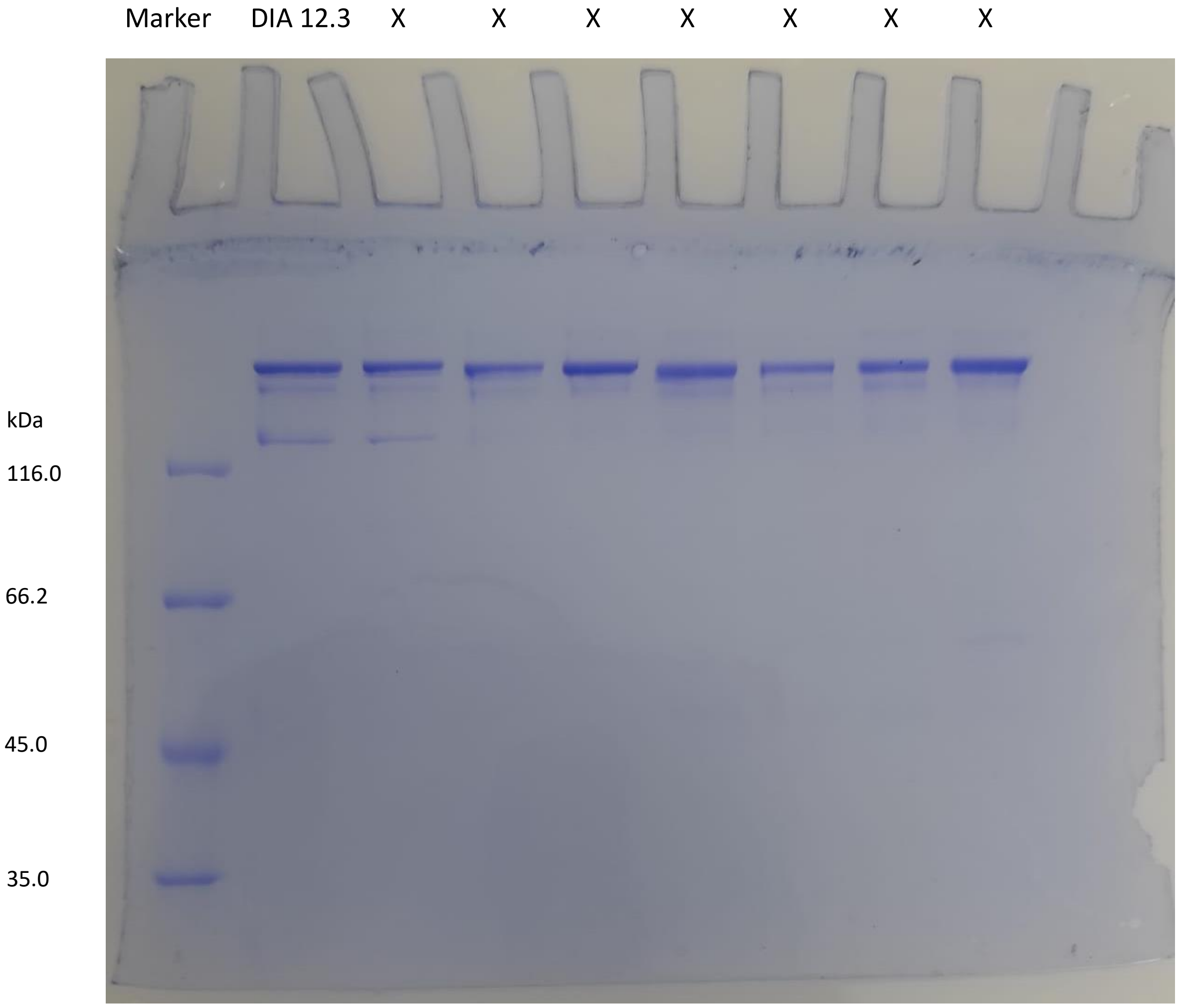

Fig 1f: SDS-Page under non-reducing conditions. The image was taken from the cell phone camera.

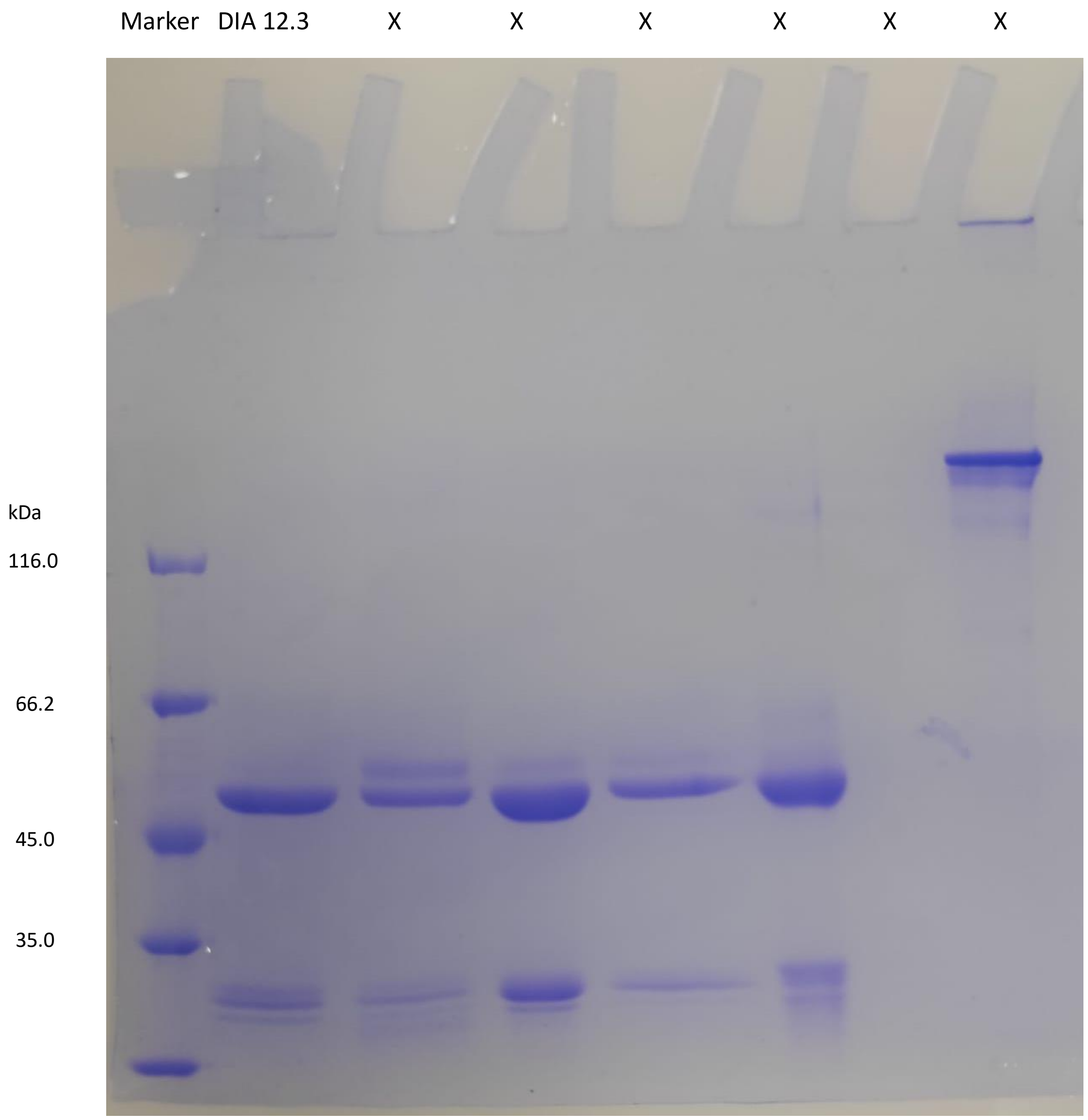

Fig 1g: SDS-Page under reducing conditions. The image was taken from the cell phone camera.
